# Public knowledge and awareness of diabetes mellitus, its risk factors, complications, and prevention methods among adults in Poland—A 2022 nationwide cross-sectional survey

**DOI:** 10.3389/fpubh.2022.1029358

**Published:** 2022-12-21

**Authors:** Kuba Sękowski, Justyna Grudziąż-Sękowska, Jarosław Pinkas, Mateusz Jankowski

**Affiliations:** School of Public Health, Centre of Postgraduate Medical Education, Warsaw, Poland

**Keywords:** diabetes mellitus, diabetes risk factors, public knowledge, prevention, preventive medicine, Poland

## Abstract

**Introduction:**

Regular monitoring of public awareness of diabetes is necessary to provide effective educational and preventive strategies. This study aimed to assess (1) public knowledge and awareness of diabetes among adults in Poland, as well as (2) to identify sociodemographic factors associated with public awareness of diabetes.

**Methods:**

This cross-sectional survey was carried out between 24 and 27 June 2022, on a non-probability random quota sample of 1,051 adults in Poland. The questionnaire included ten questions related to the awareness of risk factors, symptoms, and complications of diabetes.

**Results:**

Among the respondents, 10.5% had diabetes and 43.8% declared that they have a history of diabetes in their family. Only 17.3% of respondents declared a good level of knowledge of diabetes. Out of 10 symptoms of diabetes analyzed in this study, high blood sugar (80.7%) and chronic fatigue (74.6%) were the most recognized. Out of 8 diabetes risk factors analyzed in this study, overweight/obesity (80.4%) and unhealthy diet (74.1%) were the most recognized diabetes risk factors, while only 22.7% of respondents indicated tobacco use. The diabetic foot was the most recognized diabetes complication (79.8%), but approximately half of the respondents indicated vision problems (56.9%), kidney damage (52.1%), or cardiovascular diseases (50.2%) as diabetes complications. Female gender, having higher education and having a family member with diabetes were the most im-portent factors associated (*p* < 0.05) with a higher level of awareness of diabetes.

**Conclusions:**

This study demonstrated insufficient public awareness of diabetes among adults in Poland. Gender and educational level were the most important factors significantly associated with the awareness of the selected aspects of diabetes, while self-reported financial situation and place of residence had none or marginal influence. The presented data manifest the importance of adopting a comprehensive education strategy regarding diabetes in Poland

## 1. Introduction

Diabetes remains one of the four most prevalent non-communicable diseases (NCDs) in the world (1–3). It results in disability and premature death while creating an increasing burden on health systems, economic development, and the wellbeing of a large proportion of the global population (4). The most common forms of diabetes are type 1 diabetes, in which complete insulin deficiency causes the destruction of the pancreatic beta cells, and type 2 diabetes, in which insulin resistance can lead to hyperglycemia (5–7). Most diabetes cases (up to 95% of diabetic patients) are type 2 diabetes (so-called insulin-independent) (6, 7).

The International Diabetes Federation (IDF) estimates that as of 2021 there were 537 million people with diabetes worldwide, and this was predicted to increase to 783 million by 2045 (8). The incidence of diabetes is more prevalent in highly developed countries, but the highest rate of increase in cases is in developing countries (9). The continuing upward trend is mainly caused by the increase in the number of diabetes patients with type 2 diabetes (10), which is attributed to population growth and aging (39.7%), increased incidence (28.5%), and the interaction of these two factors (31.8%) (11). It is widely believed that the main cause of type 2 diabetes is a high-energy Western-style diet combined with a sedentary lifestyle, which underlines the role of lifestyle as the most important risk factor for type 2 diabetes (12).

Poland is a European Union (EU) member state with a high diabetes burden (13, 14). The prevalence of diabetes in Poland is estimated at 8% of the population (14). The prevalence of diabetes in Poland is significantly higher than in other EU (mean 6.3% of the population), and it is estimated that the prevalence of diabetes in Poland will rise to 11% in 2040 (15).

According to the Polish National Health Fund (a public payer in the universal health insurance system in Poland), most of the patients with diabetes who visited a doctor were females (55.1%), and the average life expectancy of diabetes patients was 15 years lower than the average for the general Polish population (16). Moreover, there are public health concerns about the under diagnosis of diabetes in Poland (14, 17). The COVID-19 pandemic may have a negative impact on the diagnosis of diabetes in Poland, as only 63% of adults in Poland had a blood sugar test during the COVID-19 pandemic (18).

Diabetes prevention, as well as disease management, requires both medications and lifestyle changes (19). Patients diagnosed with diabetes should be actively involved in disease management, as a high level of compliance may significantly in-crease the quality of life and prevent/delay long-term diabetes complications (20). The level of patients' knowledge of diabetes plays an important role in the self-management of the disease. It is considered that patients with good disease knowledge have a better understanding of the nature and consequences of diabetes and are less prone to various complications and severe exacerbations of diabetes (21, 22). Both Polish and internationally recognized standards for the treatment of diabetes emphasize that all patients should receive diabetes education and self-management training and support (23, 24). In Poland, diabetes screening is carried out as a part of general screening program, without separated program addressed to high-risk populations.

Early detection of diabetes requires both health care practices and patients' engagement (interest) based on their perception of this disease (individual health literacy level) (25). The level of health literacy affects people's decisions and actions, which includes the ability to choose and access the appropriate form of health care (26). Thus, public knowledge and awareness of diabetes reduce the gaps in diabetes under diagnosis as well as prevent long-term complications among patients with a diabetes diagnosis. Regular monitoring of public awareness of diabetes is necessary to provide effective educational and preventive strategies.

Therefore, this study aimed to assess (1) public knowledge and awareness of diabetes among adults in Poland, with a particular emphasis on diabetes risk factors, complications, and prevention methods, as well as (2) to identify sociodemographic factors associated with public awareness of diabetes symptoms and risk factors.

## 2. Materials and methods

### 2.1. Study design and population

This cross-sectional survey was carried out between 24 and 27 June 2022, on a non-probability random quota sample of 1,051 adults in Poland. Data were collected using a dedicated IT system (online panel) developed by the specialized poll company in Poland (The Nationwide Research Panel Ariadna) on behalf of the authors that pro-vide the scientific context of the study (27). A computer-assisted web interview (CAWI) method was used. Respondents were randomly selected from the dataset of 110,000 individual users of the Nationwide Research Panel Ariadna (27). Quota sampling was based on the stratification model (gender; age; place of residence) adjusted to the demographic characteristics of the Polish population according to the reports presented by the Central Statistical Office of the Republic of Poland. A similar research methodology was used in previous studies (28, 29).

The study protocol was reviewed and approved by the Ethical Review Board at the Centre of Postgraduate Medical Education, Warsaw, Poland (No. 70/2022; date of approval: 08 June 2022).

### 2.2. Questionnaire and measures

The research tool was a questionnaire developed for the purpose of this study. In preparation for the questionnaire, the previously published studies on diabetes awareness were analyzed. A particular emphasis was given to studies that used Diabetic Knowledge Questionnaire (DKQ24) (30) and Diabetes Knowledge Test (DKT) questionnaire (31). A particular emphasis was given to studies that used Diabetic Knowledge Questionnaire (DKQ24) (30) and Diabetes Knowledge Test (DKT) questionnaire (31). The questionnaire included ten questions related to the awareness of risk factors, symptoms, and complications of diabetes, as well as questions regarding the diagnosis of diabetes by a doctor and the history of diabetes in the family. Questions also addressed the personal characteristics of the respondents.

#### 2.2.1. Awareness of diabetes symptoms

Respondents were asked about their awareness of the symptoms of diabetes, using the question: “What do you think are the symptoms of diabetes (please select all that apply)?” With ten mutually non-exclusive answers. Respondents were asked to select “yes” or “no” for each answer choice.

#### 2.2.2. Awareness of the risk factors for diabetes

Respondents were asked about their awareness of the risk factors for diabetes, using the question: “What do you think are the risk factors for diabetes (please select all that apply)?” With eight mutually non-exclusive answers. Respondents were asked to select “yes” or “no” for each answer choice.

#### 2.2.3. Awareness of diabetes prevention methods

Respondents were asked about their awareness of the diabetes prevention methods, using the question: “What do you think are diabetes prevention methods (please select all that apply)?” With five mutually non-exclusive answers.

#### 2.2.4. Awareness of diabetes complications

Respondents were asked about their awareness of diabetes complications, using the question: “What do you think are diabetes complications (please select all that apply)?” With six mutually non-exclusive answers.

Moreover, respondents were asked about their health status - “Has a doctor ever told you that you have diabetes?” (yes/no). Respondents who said yes, were asked about the type of diabetes diagnosed by a doctor (type 1 diabetes; type 2 diabetes; gestational diabetes; I do not know). Also, a question on the history of diabetes in the family was addressed.

### 2.3. Data analysis

The data were analyzed with SPSS software version 28 (IBM Corp, Armonk, NY, USA). The distribution of categorical variables was shown by frequencies and proportions. Cross-tabulations and chi-squared tests were used to compare categorical variables.

Associations between personal characteristics [(1) gender, (2) age group, (3) having higher education, (4) marital status, (5) having children, (6) place of residence, (7) a number of household members, (8) occupational status, (9) self-reported financial situation, (10) having diabetes, (11) history of diabetes in the family] and awareness of (1) diabetes symptoms and (2) risk factors for diabetes were analyzed using multivariable logistic regression models. The strength of association was measured by the odds ratio (OR) and 95% confidence intervals (95% CI). The level of statistical significance was set at *p* < 0.05.

## 3. Results

### 3.1. Characteristics of the study population

Data were obtained from 1,051 individuals aged 18–85 years, 53.3% were females (Table 1). Most of the respondents were married (49.5%), 42.8% had higher education and one-third (32.3%) lived in rural areas. Among the respondents, 10.5% had diabetes. Out of 110 respondents with diabetes, 56.4% had type 2 diabetes, 15.5% had type 1 diabetes, and 11.8% had gestational diabetes. Among the respondents with diabetes, 16.4% were unaware of the type of diabetes they were diagnosed with. Out of all respondents, 43.8% declared that they have a history of diabetes in their family, wherein most of the respondents were not aware of the type of diabetes in their family (21.6% of all the respondents), 19% had a history of type 2 diabetes in the family, 6.5% type 1 diabetes and 1.5% reported gestational diabetes. Characteristics of the study population are presented in Table 1.

**Table 1:** Characteristics of the study population (*n* = 1,051).

**Variable**	**Total sample *n =* 1,051**	
**Overall**	***n***	**%**
**Gender**		
Female	560	53.3
Male	491	46.7
**Age (years)**		
18–29	226	21.5
30–39	209	19.9
40–49	190	18.1
50–59	202	19.2
60+	224	21.3
**Educational level**		
Primary	28	2.7
Vocational	109	10.4
Secondary	464	44.1
Higher	450	42.8
**Marital status**		
Single	250	23.8
Married	520	49.5
Informal relationship	164	15.6
Divorced/widowed	117	11.1
**Having children**		
Yes	643	61.2
No	408	38.8
**Place of residence**		
Rural	339	32.3
City below 20,000 residents	122	11.6
City from 20,000 to 99,999 residents	237	22.5
City from 100,000 to 499,999 residents	200	19.0
City above 500,000 residents	153	14.6
**Number of household members**		
1	159	15.1
2 or more	892	84.9
**Occupational status**		
Active	663	63.1
Passive	388	36.9
**Self-reported financial situation**		
Good	401	38.2
Moderate	406	38.6
Bad	244	23.2
**Having diabetes**		
Yes	110	10.5
No	941	89.5
**History of diabetes in the family**		
Yes	460	43.8
No	591	56.2

### 3.2. Respondents' knowledge of diabetes

Most of the respondents declared a moderate (46.3%) level of knowledge of diabetes and only 17.3% of respondents declared rather good or very good knowledge of diabetes (Table 2). Out of 10 symptoms of diabetes analyzed in this study, high blood sugar (80.7%) and chronic fatigue, feeling sleepy during the day (74.6%) were the most recognized symptoms. Most of the respondents (57.4%) were aware that polydipsia is a symptom of diabetes, but only 42% of respondents indicated polyuria as a symptom of diabetes (Table 2). Persistent skin itching (19.7%) and increased risk of infections (22.6%) were the least recognized symptoms of diabetes. Out of 8 diabetes risk factors analyzed in this study, overweight/obesity (80.4%), unhealthy diet (74.1%) and genetic predisposition (69.5%) were the most recognized diabetes risk factors (Table 2). Tobacco use (22.7%) was the least recognized risk factor for diabetes. Approximately three quarters of respondents were aware that limited consumption of carbohydrates (sugars) in the diet (77.1%), weight reduction in overweight or obese people (75.1%) or regular physical activity (73%) are diabetes prevention methods. Diabetic foot was the most recognized diabetes complication (79.8%). More than half of respondents were aware that diabetes may lead to vision problems (56.9%), kidney damage (52.1%) or cardiovascular diseases (50.2%). Details are presented in Table 2.

**Table 2:** Respondents' knowledge of diabetes (*n* = 1,051).

**Variable**	**Overall** **(*n =* 1,051)**	
	***n***	**%**
**Self-reported level of knowledge on diabetes**		
Very bad	80	7.6
Rather bad	302	28.7
Moderate	487	46.3
Rather good	137	13.0
Very good	45	4.3
**What do you think are the symptoms of diabetes? (multiple-choice question; positive answers)**		
High blood sugar (hyperglycemia)	848	80.7
Polyuria	441	42.0
Increased thirst or a feeling of dry mouth (polydipsia)	603	57.4
Unexpected excessive weight loss	310	29.5
Slow-healing wounds	615	58.5
Deterioration of vision (e.g., blurred vision)	539	51.3
Numbness and/or tingling of hands or feet	271	25.8
Increased risk of infections (e.g., bacterial or fungal skin infections)	238	22.6
Persistent skin itching	207	19.7
Chronic fatigue, feeling sleepy during the day	784	74.6
**What do you think are the risk factors for diabetes? (multiple-choice question; positive answers)**		
Excessive alcohol consumption	326	31.0
Smoking cigarettes/tobacco	239	22.7
Overweight/obesity	845	80.4
Low physical activity level (e.g., sedentary lifestyle)	649	61.8
Unhealthy diet (e.g., eating highly processed foods, high amounts of fatty foods, low fiber intake)	779	74.1
Arterial hypertension	311	29.6
Age > 40–45 years	301	28.6
Genetic predisposition (history of diabetes in the family)	730	69.5
**What do you think are diabetes prevention methods? (multiple-choice question; positive answers)**		
Regular physical activity	767	73.0
Limited intake of fats in the diet	569	54.1
Limited consumption of carbohydrates (sugars) in the diet	810	77.1
Limited alcohol consumption	471	44.8
Weight reduction in overweight or obese people	789	75.1
**What do you think are diabetes complications? (multiple-choice question; positive answers)**		
Cardiovascular diseases such as heart attack or stroke	528	50.2
Kidney damage	548	52.1
Vision problems/loss of vision	598	56.9
Limb amputation (e.g., Leg amputation)	708	67.4
Diabetic foot	839	79.8
Damage to the nervous system leading to sensory disturbances	311	29.6

There were statistically significant differences in the percentage of respondents who correctly indicated diabetes symptoms by gender, age, educational level, marital status, having children, and place of residence. Moreover, respondents who were diagnosed with diabetes or those with history of diabetes in the family more often correctly indicated diabetes symptoms (Table 3). There were significant differences (*p* < 0.05) in the percentage of respondents who correctly indicated diabetes risk factors depending on the gender, age, educational level, having children, number of household members occupational status (Table 4). Those who had diabetes more often indicated overweight/obesity as diabetes risk factors. Moreover, the percentage of respondents who correctly indicated diabetes risk factor was higher among those respondents who had history of diabetes in the family (Table 4).

**Table 3:** Awareness of diabetes symptoms by sociodemographic factors (*n* = 1,051).

	**Diabetes symptoms - percentage of respondents who answered “yes” by sociodemographic factors**									
**Variable**	**High blood sugar**		**Polyuria**		**Increased thirst or a feeling of dry mouth (polydypsia)**		**Unexpected excessive weight loss**		**Slow-healing wounds**	
	***n* (%)**	***p***	***n* (%)**	***p***	***n* (%)**	***p***	***n* (%)**	***p***	***n* (%)**	***p***
**Gender**										
Female	478 (85.4)	**< 0.001**	267 (47.7)	**< 0.001**	279 (67.7)	**< 0.001**	197 (35.2)	**< 0.001**	374 (66.8)	**< 0.001**
Male	370 (75.4)		174 (35.4)		224 (45.6)		113 (23.0)		241 (49.1)	
**Age (years)**										
18–29	162 (71.7)	**< 0.001**	75 (33.2)	**0.048**	108 (47.8)	**< 0.001**	65 (28.8)	0.1	94 (41.6)	**< 0.001**
30–39	152 (72.7)		92 (44.0)		111 (53.1)		55 (26.3)		110 (52.6)	
40–49	157 (82.6)		81 (42.6)		108 (56.8)		46 (24.2)		115 (60.5)	
50–59	176 (87.1)		90 (44.6)		129 (63.9)		66 (32.7)		137 (67.8)	
60+	201 (89.7)		103 (46.0)		147 (65.6)		78 (34.8)		159 (71.0)	
**Educational level**										
Primary	19 (67.9)	**0.04**	10 (35.7)	**0.02**	13 (46.4)	0.05	3 (10.7)	**0.01**	14 (50.0)	0.3
Vocational	80 (73.4)		37 (33.9)		55 (50.5)		23 (21.1)		57 (52.3)	
Secondary	375 (80.8)		182 (39.2)		257 (55.4)		136 (29.3)		271 (58.4)	
Higher	374 (83.1)		212 (47.1)		278 (61.8)		148 (32.9)		273 (60.7)	
**Marital status**										
Single	182 (72.8)	**< 0.001**	103 (41.2)	0.7	136 (54.4)	0.2	66 (26.4)	0.6	121 (48.4)	**< 0.001**
Married	431 (82.9)		223 (42.9)		304 (58.5)		155 (29.8)		322 (61.9)	
Informal relationship	130 (79.3)		63 (38.4)		88 (53.7)		52 (31.7)		94 (57.3)	
Divorced/widowed	105 (89.7)		52 (44.4)		75 (64.1)		37 (31.6)		78 (66.7)	
**Having children**										
Yes	543 (84.4)	**< 0.001**	280 (43.5)	0.2	394 (61.3)	**0.001**	202 (31.4)	0.09	412 (64.1)	**< 0.001**
No	305 (74.8)		161 (39.5)		209 (51.2)		108 (26.5)		203 (49.8)	
**Place of residence**										
Rural	269 (79.4)	0.2	123 (36.3)	0.09	178 (52.5)	0.3	83 (24.5)	0.08	186 (54.9)	0.3
City below 20,000 residents	104 (85.2)		52 (42.6)		75 (61.5)		34 (27.9)		72 (59.0)	
City from 20,000 to 99,999 residents	182 (76.8)		102 (43.0)		139 (58.6)		83 (35.0)		142 (59.9)	
City from 100,000 to 499,999 residents	165 (82.5)		90 (45.0)		119 (59.5)		61 (30.5)		116 (58.0)	
City above 500,000 residents	128 (83.7)		74 (48.4)		92 (60.1)		49 (32.0)		99 (64.7)	
**Number of household members**										
1	125 (78.6)	0.5	70 (44.0)	0.6	101 (63.5)	0.09	49 (30.8)	0.7	98 (61.6)	0.4
2 or more	723 (81.1)		371 (41.6)		502 (56.3)		261 (29.3)		517 (58.0)	
**Occupational status**										
Active	529 (79.8)	0.3	280 (42.2)	0.8	371 (56.0)	0.2	197 (29.7)	0.8	382 (57.6)	0.4
Passive	319 (82.2)		161 (41.5)		232 (59.8)		113 (29.1)		233 (60.1)	
**Self-reported financial situation**										
Good	326 (81.3)	0.8	178 (44.4)	0.5	221 (55.1)	0.5	129 (32.2)	0.3	237 (59.1)	0.5
Moderate	329 (81.0)		165 (40.6)		239 (58.9)		111 (27.3)		243 (59.9)	
Bad	193 (79.1)		98 (40.2)		143 (58.6)		70 (28.7)		135 (55.3)	
**Having diabetes**										
Yes	98 (89.1)	**0.02**	63 (57.3)	**< 0.001**	83 (75.5)	**< 0.001**	47 (42.7)	**0.001**	77 (70.0)	**0.01**
No	750 (79.7)		378 (40.2)		520 (55.3)		263 (27.9)		538 (57.2)	
**History of diabetes in the family**										
Yes	391 (85.0)	**0.002**	221 (48.0)	**< 0.001**	299 (65.0)	**< 0.001**	161 (35.0)	**< 0.001**	312 (67.8)	**< 0.001**
No	457 (77.3)		220 (37.2)		304 (51.4)		149 (25.2)		303 (51.3)	
**Gender**										
Female	308 (55.0)	**0.01**	149 (26.6)	0.5	156 (27.9)	**< 0.001**	142 (25.4)	**< 0.001**	458 (81.8)	**< 0.001**
Male	231 (47.0)		122 (24.8)		82 (16.7)		65 (13.2)		326 (66.4)	
**Age (years)**										
18–29	86 (38.1)	**< 0.001**	64 (28.3)	0.5	42 (18.6)	0.3	32 (14.2)	**0.003**	153 (67.7)	**0.02**
30–39	103 (49.3)		60 (28.7)		54 (25.8)		33 (15.8)		149 (71.3)	
40–49	102 (53.7)		42 (22.1)		39 (20.5)		34 (17.9)		148 (77.9)	
50–59	120 (59.4)		52 (25.7)		47 (23.3)		55 (27.2)		163 (80.7)	
60+	128 (57.1)		53 (23.7)		56 (25.0)		53 (23.7)		171 (76.3)	
**Educational level**										
Primary	14 (50.0)	**0.01**	9 (32.1)	**0.006**	7 (25.0)	**< 0.001**	2 (7.1)	**0.02**	17 (60.7)	0.1
Vocational	51 (46.8)		18 (16.5)		11 (10.1)		14 (12.8)		74 (67.9)	
Secondary	217 (46.8)		107 (23.1)		80 (17.2)		86 (18.5)		349 (75.2)	
Higher	257 (57.1)		137 (30.4)		140 (31.1)		105 (23.3)		344 (76.4)	
**Marital status**										
Single	110 (44.0)	**0.046**	72 (28.8)	0.1	53 (21.2)	0.8	45 (18.0)	0.1	177 (70.8)	0.4
Married	283 (54.4)		117 (22.5)		124 (23.8)		109 (21.0)		393 (75.6)	
Informal relationship	82 (50.0)		48 (29.3)		35 (21.3)		24 (14.6)		122 (74.4)	
Divorced/widowed	64 (54.7)		34 (29.1)		26 (22.2)		29 (24.8)		92 (78.6)	
**Having children**										
Yes	354 (55.1)	**0.002**	156 (24.3)	0.2	151 (23.5)	0.4	141 (21.9)	**0.02**	498 (77.4)	**0.008**
No	185 (45.3)		115 (28.2)		87 (21.3)		66 (16.2)		286 (70.1)	
**Place of residence**										
Rural	165 (48.7)	0.7	73 (21.5)	**0.01**	57 (16.8)	**0.02**	51 (15.0)	**0.04**	246 (72.6)	0.7
City below 20,000 residents	65 (53.3)		27 (22.1)		27 (22.1)		29 (23.8)		91 (74.6)	
City from 20,000 to 99,999 residents	125 (52.7)		71 (30.0)		58 (24.5)		59 (24.9)		182 (76.8)	
City from 100,000 to 499,999 residents	108 (54.0)		47 (23.5)		56 (28.0)		37 (18.5)		146 (73.0)	
City above 500,000 residents	76 (49.7)		53 (34.6)		40 (26.1)		31 (20.3)		119 (77.8)	
**Number of household members**										
1	79 (49.7)	0.7	44 (27.7)	0.6	40 (25.2)	0.4	37 (23.3)	0.2	120 (75.5)	0.8
2 or more	460 (51.6)		227 (25.4)		198 (22.2)		170 (19.1)		664 (74.4)	
**Occupational status**										
Active	343 (51.7)	0.7	173 (26.1)	0.8	155 (23.4)	0.5	126 (19.0)	0.5	490 (73.9)	0.5
Passive	196 (50.5)		98 (25.3)		83 (21.4)		81 (20.9)		294 (75.8)	
**Self-reported financial situation**										
Good	206 (51.4)	0.6	102 (25.4)	0.8	92 (22.9)	0.5	71 (17.7)	0.4	299 (74.6)	0.3
Moderate	214 (52.7)		109 (26.8)		97 (23.9)		84 (20.7)		311 (76.6)	
Bad	119 (48.8)		60 (24.6)		49 (20.1)		52 (21.3)		174 (71.3)	
**Having diabetes**										
Yes	79 (71.8)	**< 0.001**	43 (39.1)	**< 0.001**	27 (24.5)	0.6	27 (24.5)	0.2	83 (75.5)	0.8
No	460 (48.9)		228 (24.2)		211 (22.4)		180 (19.1)		701 (74.5)	
**History of diabetes in the family**										
Yes	276 (60.0)	**< 0.001**	145 (31.5)	**< 0.001**	124 (27.0)	**0.003**	106 (23.0)	**0.02**	374 (81.3)	**< 0.001**
No	263 (44.5)		126 (21.3)		114 (19.3)		101 (17.1)		410 (69.4)	

The bold values present results that meet the statistical significance requirement set at *p* < 0.05.

**Table 4:** Awareness of risk factors for diabetes by sociodemographic factors (*n* = 1,051).

	**Risk factors for diabetes - percentage of respondents who answered “yes” by sociodemographic factors**											
**Variable**	**Excessive alcohol consumption**		**Smoking cigarettes/tobacco**		**Overweight/obesity**		**Low physical activity level**		**Unhealthy diet**		**Genetic predisposition**	
	***n* (%)**	***p***	***n* (%)**	***p***	***n* (%)**	***p***	***n* (%)**	***p***	***n* (%)**	***p***	***n* (%)**	***p***
**Gender**												
Female	170 (30.4)	0.6	139 (24.8)	0.09	471 (84.1)	**0.001**	378 (67.5)	**< 0.001**	453 (80.9)	**< 0.001**	452 (80.7)	**< 0.001**
Male	156 (31.8)		100 (20.4)		374 (76.2)		271 (55.2)		326 (66.4)		278 (56.6)	
**Age (years)**												
18–29	67 (29.6)	0.1	51 (22.6)	0.2	163 (72.1)	**< 0.001**	131 (58.0)	0.2	152 (67.3)	**0.02**	136 (60.2)	**< 0.001**
30–39	73 (34.9)		47 (22.5)		159 (76.1)		136 (65.1)		152 (72.7)		139 (66.5)	
40–49	66 (34.7)		49 (25.8)		150 (78.9)		108 (56.8)		141 (74.2)		130 (68.4)	
50–59	64 (31.7)		53 (26.2)		182 (90.1)		133 (65.8)		153 (75.7)		157 (77.7)	
60+	56 (25.0)		39 (17.4)		191 (85.3)		141 (62.9)		181 (80.8)		168 (75.0)	
**Educational level**												
Primary	3 (10.7)	**0.002**	6 (21.4)	**< 0.001**	18 (64.3)	**< 0.001**	18 (64.3)	**< 0.001**	20 (71.4)	**< 0.001**	14 (50.0)	**0.03**
Vocational	21 (19.3)		13 (11.9)		74 (67.9)		49 (45.0)		63 (57.8)		70 (64.2)	
Secondary	147 (31.7)		92 (19.8)		363 (78.2)		262 (56.5)		343 (73.9)		319 (68.8)	
Higher	155 (34.4)		128 (28.4)		390 (86.7)		320 (71.1)		353 (78.4)		327 (72.7)	
**Marital status**												
Single	72 (28.8)	0.4	49 (19.6)	0.2	194 (77.6)	0.5	144 (57.6)	0.5	174 (69.6)	0.1	159 (63.6)	0.09
Married	162 (31.2)		128 (24.6)		420 (80.8)		327 (62.9)		386 (74.2)		369 (71.0)	
Informal relationship	59 (36.0)		41 (25.0)		132 (80.5)		103 (62.8)		124 (75.6)		114 (69.5)	
Divorced/widowed	33 (28.2)		21 (17.9)		99 (84.6)		75 (64.1)		95 (81.2)		88 (75.2)	
**Having children**												
Yes	202 (31.4)	0.7	157 (24.4)	0.1	538 (83.7)	**< 0.001**	407 (63.3)	0.2	500 (77.8)	**< 0.001**	467 (72.6)	**0.005**
No	124 (30.4)		82 (20.1)		307 (75.2)		242 (59.3)		279 (68.4)		263 (64.5)	
**Place of residence**												
Rural	103 (30.4)	0.5	76 (22.4)	0.4	266 (78.5)	0.4	198 (58.4)	0.5	251 (74.0)	0.7	218 (64.3)	0.06
City below 20,000 residents	46 (37.7)		33 (27.0)		98 (80.3)		76 (62.3)		89 (73.0)		85 (69.7)	
City from 20,000 to 99,999 residents	68 (28.7)		46 (19.4)		186 (78.5)		145 (61.2)		179 (75.5)		173 (73.0)	
City from 100,000 to 499,999 residents	60 (30.0)		44 (22.0)		165 (82.5)		129 (64.5)		142 (71.0)		137 (68.5)	
City above 500,000 residents	49 (32.0)		40 (26.1)		130 (85.0)		101 (66.0)		118 (77.1)		117 (76.5)	
**Number of household members**												
1	35 (22.0)	**0.008**	27 (17.0)	0.06	125 (78.6)	0.5	90 (56.6)	0.1	111 (69.8)	0.2	111 (69.8)	0.9
2 or more	291 (32.6)		212 (23.8)		720 (80.7)		559 (62.7)		668 (74.9)		619 (69.4)	
**Occupational status**												
Active	230 (34.7)	**< 0.001**	169 (25.5)	**0.005**	525 (79.2)	0.2	410 (61.8)	0.9	478 (72.1)	0.05	450 (67.9)	0.1
Passive	96 (24.7)		70 (18.0)		320 (82.5)		239 (61.6)		301 (77.6)		280 (72.2)	
**Self-reported financial situation**												
Good	126 (31.4)	0.8	79 (19.7)	0.2	331 (82.5)	0.2	247 (61.6)	0.9	312 (77.8)	0.1	282 (70.3)	0.9
Moderate	121 (29.8)		100 (24.6)		327 (80.5)		249 (61.3)		293 (72.2)		281 (69.2)	
Bad	79 (32.4)		60 (24.6)		187 (76.6)		153 (62.7)		174 (71.3)		167 (68.4)	
**Having diabetes**												
Yes	34 (30.9)	0.9	19 (17.3)	0.1	97 (88.2)	**0.03**	76 (69.1)	0.09	87 (79.1)	0.2	83 (75.5)	0.1
No	292 (31.0)		220 (23.4)		748 (79.5)		573 (60.9)		692 (73.5)		647 (68.8)	
**History of diabetes in the family**												
Yes	165 (35.9)	**0.003**	114 (24.8)	0.2	380 (82.6)	0.1	319 (69.3)	**< 0.001**	364 (79.1)	**0.001**	359 (78.0)	**< 0.001**
No	161 (27.2)		125 (21.2)		465 (78.7)		330 (55.8)		415 (70.2)		371 (62.8)	

The bold values present results that meet the statistical significance requirement set at *p* < 0.05.

In general, the percentage of respondents who correctly indicated diabetes complications was higher among females (Table 5). Moreover, public awareness of diabetes complications increased with the age (Table 5). The percentage of respondents who correctly indicated diabetes complications was higher among those respondents who had higher education (Table 5). Respondents who had children more often indicated vision problems, limb amputation, and diabetic foot as a diabetes complication (*p* < 0.05). In general, the percentage of respondents who correctly indicated symptoms of diabetes increased with the size of the place of residence (Table 5). There were no statistically significant differences in the percentage of respondents who correctly indicated diabetes complications by self-reported financial situation or number of household members (Table 5). Individuals diagnosed with diabetes or those with a history of diabetes in the family were more aware of diabetes complications (Table 5).

**Table 5:** Awareness of diabetes complications by sociodemographic factors (*n* = 1,051).

	**Diabetes complications - percentage of respondents who answered “yes” by sociodemographic factors**											
**Variable**	**Cardiovascular diseases**		**Kidney damage**		**Vision problems/loss of vision**		**Limb amputation**		**Diabetic foot**		**Damage to the nervous system**	
	***n* (%)**	***p***	***n* (%)**	***p***	***n* (%)**	***p***	***n* (%)**	***p***	***n* (%)**	***p***	***n* (%)**	***p***
**Gender**												
Female	298 (53.2)	**0.04**	326 (58.2)	**< 0.001**	353 (63.0)	**< 0.001**	414 (73.9)	**< 0.001**	486 (86.8)	**< 0.001**	185 (33.0)	**0.009**
Male	230 (46.8)		222 (45.2)		245 (49.9)		294 (59.9)		353 (71.9)		126 (25.7)	
**Age (years)**												
18–29	107 (47.3)	0.5	101 (44.7)	**0.01**	81 (35.8)	**< 0.001**	115 (50.9)	**< 0.001**	156 (69.0)	**< 0.001**	63 (27.9)	**< 0.001**
30–39	115 (55.0)		98 (46.9)		113 (54.1)		140 (67.0)		155 (74.2)		78 (37.3)	
40–49	96 (50.5)		108 (56.8)		114 (60.0)		124 (65.3)		150 (78.9)		50 (26.3)	
50–59	104 (51.5)		112 (55.4)		141 (69.8)		161 (79.7)		171 (84.7)		73 (36.1)	
60+	106 (47.3)		129 (57.6)		149 (66.5)		168 (75.0)		207 (92.4)		47 (21.0)	
**Educational level**												
Primary	14 (50.0)	**< 0.001**	11 (39.3)	**< 0.001**	9 (32.1)	**< 0.001**	12 (42.9)	**< 0.001**	18 (64.3)	**< 0.001**	9 (32.1)	**< 0.001**
Vocational	40 (36.7)		47 (43.1)		49 (45.0)		65 (59.6)		75 (68.8)		13 (11.9)	
Secondary	218 (47.0)		223 (48.1)		253 (54.5)		298 (64.2)		362 (78.0)		124 (26.7)	
Higher	256 (56.9)		267 (59.3)		287 (63.8)		333 (74.0)		384 (85.3)		165 (36.7)	
**Marital status**												
Single	127 (50.8)	0.8	125 (50.0)	0.8	121 (48.4)	**0.004**	141 (56.4)	**< 0.001**	182 (72.8)	**0.002**	80 (32.0)	0.8
Married	254 (48.8)		273 (52.5)		311 (59.8)		361 (69.4)		421 (81.0)		150 (28.8)	
Informal relationship	88 (53.7)		85 (51.8)		89 (54.3)		114 (69.5)		131 (79.9)		48 (29.3)	
Divorced/widowed	59 (50.4)		65 (55.6)		77 (65.8)		92 (78.6)		105 (89.7)		33 (28.2)	
**Having children**												
Yes	325 (50.5)	0.8	342 (53.2)	0.4	403 (62.7)	**< 0.001**	467 (72.6)	**< 0.001**	542 (84.3)	**< 0.001**	184 (28.6)	0.4
No	203 (49.8)		206 (50.5)		195 (47.8)		241 (59.1)		297 (72.8)		127 (31.1)	
**Place of residence**												
Rural	166 (49.0)	0.3	148 (43.7)	**0.005**	171 (50.4)	**0.03**	206 (60.8)	**0.01**	243 (71.7)	**< 0.001**	87 (25.7)	0.3
City below 20,000 residents	67 (54.9)		68 (55.7)		68 (55.7)		81 (66.4)		101 (82.8)		42 (34.4)	
City from 20,000 to 99,999 residents	127 (53.6)		131 (55.3)		141 (59.5)		162 (68.4)		199 (84.0)		76 (32.1)	
City from 100,000 to 499,999 residents	101 (50.5)		111 (55.5)		119 (59.5)		145 (72.5)		163 (81.5)		57 (28.5)	
City above 500,000 residents	67 (43.8)		90 (58.8)		99 (64.7)		114 (74.5)		133 (86.9)		49 (32.0)	
**Number of household members**												
1	75 (47.2)	0.4	86 (54.1)	0.6	89 (56.0)	0.8	106 (66.7)	0.8	133 (83.6)	0.2	48 (30.2)	0.9
2 or more	453 (50.8)		462 (51.8)		509 (57.1)		602 (67.5)		706 (79.1)		263 (29.5)	
**Occupational status**												
Active	336 (50.7)	0.7	342 (51.6)	0.6	377 (56.9)	0.9	451 (68.0)	0.6	514 (77.5)	**0.02**	206 (31.1)	0.2
Passive	192 (49.5)		206 (53.1)		221 (57.0)		257 (66.2)		325 (83.8)		105 (27.1)	
**Self-reported financial situation**												
Good	193 (48.1)	0.4	215 (53.6)	0.5	228 (56.9)	0.9	272 (67.8)	0.6	323 (80.5)	0.8	118 (29.4)	0.9
Moderate	205 (50.5)		203 (50.0)		228 (56.2)		278 (68.5)		324 (79.8)		122 (30.0)	
Bad	130 (53.3)		130 (53.3)		142 (58.2)		158 (64.8)		192 (78.7)		71 (29.1)	
**Having diabetes**												
Yes	61 (55.5)	0.2	62 (56.4)	0.3	81 (73.6)	**< 0.001**	83 (75.5)	0.06	101 (91.8)	**< 0.001**	45 (40.9)	**0.006**
No	467 (49.6)		486 (51.6)		517 (54.9)		625 (66.4)		738 (78.4)		266 (28.3)	
**History of diabetes in the family**												
Yes	270 (58.7)	**< 0.001**	261 (56.7)	**0.008**	289 (62.8)	**< 0.001**	333 (72.4)	**0.002**	385 (83.7)	**0.006**	157 (34.1)	**0.004**
No	258 (43.7)		287 (48.6)		309 (52.3)		375 (63.5)		454 (76.8)		154 (26.1)	

The bold values present results that meet the statistical significance requirement set at *p* < 0.05.

The percentage of respondents who correctly indicated diabetes prevention methods was higher among females (Table 6). Moreover, public awareness of diabetes prevention methods increased with age and educational level (Table 6). Those who had ever been married as well as those who had children more often correctly indicated diabetes prevention methods. The percentage of respondents who were aware that limited sugar intake and weight reduction in overweight/obese individuals are diabetes prevention methods was higher among those who lived in the largest cities (*p* < 0.05). Respondents who lived with at least one person more often declared that a limited intake of sugar is a diabetes prevention method (*p* < 0.05). Moreover, those with passive occupational status more often declared limited sugar intake as a diabetes prevention method (*p* < 0.05). Individuals diagnosed with diabetes or those with a history of diabetes in the family were more aware of diabetes prevention methods. There were no differences (*p* > 0.05) in public awareness of diabetes prevention methods de-pending on financial status or having a diagnosis of diabetes.

**Table 6:** Awareness of diabetes prevention methods by sociodemographic factors (*n* = 1,051).

	**Diabetes prevention methods - percentage of respondents who answered “yes” by sociodemographic factors**									
**Variable**	**Regular physical activity**		**Limited intake of fats in the diet**		**Limited consumption of carbohydrates (sugars) in the diet**		**Limited alcohol consumption**		**Weight reduction in overweight or obese people**	
	***n* (%)**	***p***	***n* (%)**	***p***	***n* (%)**	**p**	***n* (%)**	***p***	***n* (%)**	***p***
**Gender**										
Female	431 (77.0)	**0.002**	330 (58.9)	**< 0.001**	459 (82.0)	**< 0.001**	267 (47.7)	**0.046**	450 (80.4)	**< 0.001**
Male	336 (68.4)		239 (48.7)		351 (71.5)		204 (41.5)		339 (69.0)	
**Age (years)**										
18–29	146 (64.6)	**0.002**	99 (43.8)	**0.003**	149 (65.9)	**< 0.001**	96 (42.5)	0.9	138 (61.1)	**< 0.001**
30–39	158 (75.6)		120 (57.4)		162 (77.5)		95 (45.5)		155 (74.2)	
40–49	130 (68.4)		97 (51.1)		142 (74.7)		83 (43.7)		136 (71.6)	
50–59	155 (76.7)		119 (58.9)		160 (79.2)		94 (46.5)		170 (84.2)	
60+	178 (79.5)		134 (59.8)		197 (87.9)		103 (46.0)		190 (84.8)	
**Educational level**										
Primary	18 (64.3)	**< 0.001**	12 (42.9)	**0.003**	18 (64.3)	**0.001**	6 (21.4)	**< 0.001**	16 (57.1)	**< 0.001**
Vocational	63 (57.8)		50 (45.9)		71 (65.1)		36 (33.0)		69 (63.3)	
Secondary	328 (70.7)		235 (50.6)		355 (76.5)		201 (43.3)		344 (74.1)	
Higher	358 (79.6)		272 (60.4)		366 (81.3)		228 (50.7)		360 (80.0)	
**Marital status**										
Single	174 (69.6)	0.4	113 (45.2)	**0.01**	170 (68.0)	**< 0.001**	101 (40.4)	0.4	171 (68.4)	**0.005**
Married	388 (74.6)		296 (56.9)		418 (80.4)		238 (45.8)		399 (76.7)	
Informal relationship	116 (70.7)		92 (56.1)		125 (76.2)		75 (45.7)		120 (73.2)	
Divorced/widowed	89 (76.1)		68 (58.1)		97 (82.9)		57 (48.7)		99 (84.6)	
**Having children**										
Yes	485 (75.4)	**0.03**	368 (57.2)	**0.01**	518 (80.6)	**< 0.001**	287 (44.6)	0.9	506 (78.7)	**< 0.001**
No	282 (69.1)		201 (49.3)		292 (71.6)		184 (45.1)		283 (69.4)	
**Place of residence**										
Rural	236 (69.6)	0.5	170 (50.1)	0.2	245 (72.3)	**0.002**	149 (44.0)	0.8	238 (70.2)	**0.02**
City below 20,000 residents	91 (74.6)		66 (54.1)		101 (82.8)		59 (48.4)		88 (72.1)	
City from 20,000 to 99,999 residents	175 (73.8)		142 (59.9)		187 (78.9)		101 (42.6)		187 (78.9)	
City from 100,000 to 499,999 residents	149 (74.5)		110 (55.0)		145 (72.5)		93 (46.5)		149 (74.5)	
City above 500,000 residents	116 (75.8)		81 (52.9)		132 (86.3)		69 (45.1)		127 (83.0)	
**Number of household members**										
1	109 (68.6)	0.2	72 (45.3)	**0.02**	122 (76.7)	0.9	61 (38.4)	0.08	119 (74.8)	0.9
2 or more	658 (73.8)		497 (55.7)		688 (77.1)		410 (46.0)		670 (75.1)	
**Occupational status**										
Active	472 (71.2)	0.09	359 (54.1)	0.9	493 (74.4)	**0.006**	305 (46.0)	0.3	489 (73.8)	0.2
Passive	295 (76.0)		210 (54.1)		317 (81.7)		166 (42.8)		300 (77.3)	
**Self-reported financial situation**										
Good	295 (73.6)	0.3	219 (54.6)	0.8	318 (79.3)	0.07	186 (46.4)	0.7	305 (76.1)	0.4
Moderate	303 (74.6)		215 (53.0)		317 (78.1)		176 (43.3)		309 (76.1)	
Bad	169 (69.3)		135 (55.3)		175 (71.7)		109 (44.7)		175 (71.7)	
**Having diabetes**										
Yes	86 (78.2)	0.2	62 (56.4)	0.6	91 (82.7)	0.1	45 (40.9)	0.4	90 (81.8)	0.08
No	681 (72.4)		507 (53.9)		719 (76.4)		426 (45.3)		699 (74.3)	
**History of diabetes in the family**										
Yes	358 (77.8)	**0.002**	282 (61.3)	**< 0.001**	375 (81.5)	**0.002**	219 (47.6)	0.1	366 (79.6)	**0.003**
No	409 (69.2)		287 (48.6)		435 (73.6)		252 (42.6)		423 (71.6)	

The bold values present results that meet the statistical significance requirement set at *p* < 0.05.

### 3.3. Factors associated with respondents' awareness of diabetes symptoms

Female gender and having higher education were the most important factors associated (*p* < 0.05) with a higher level of awareness of most of the diabetes symptoms (Table 7). Older respondents were more aware (*p* < 0.05) that high blood sugar, polyuria, polydipsia, slow-healing wounds, deterioration of vision, and chronic fatigue are the symptoms of diabetes (Table 7). Respondents who lived in cities from 20,000 to 99,999 residents were more likely to indicate unexpected excessive weight loss, numbness/tingling of hands or feet, and persistent skin itching as diabetes symptoms. Respondents who were diagnosed with diabetes were more likely (*p* < 0.05) to indicate polyuria, polydipsia, unexpected excessive weight loss, deterioration of vision, and numbness/tingling of hands or feet as diabetes symptoms. In general, respondents with a history of diabetes in the family had a higher level of knowledge of diabetes symptoms (Table 7). In the multivariable logistic regression model, there was no influence (*p* > 0.05) of (1) marital status, (2) having children, (3) number of household members, (4) occupational status, and (5) financial situation on the respondents' awareness of diabetes symptoms.

**Table 7:** Factors associated with awareness of diabetes symptoms among adults in Poland (*n* = 1,051)—multivariable logistic regression model.

	**Factors associated with awareness of diabetes symptoms among adults in Poland**									
**Variable**	**High blood sugar**		**Polyuria**		**Increased thirst or a feeling of dry mouth (polydipsia)**		**Unexpected excessive weight loss**		**Slow–healing wounds**	
	**OR (95%CI)**	***p***	**OR (95%CI)**	***p***	**OR (95%CI)**	***p***	**OR (95%CI)**	***p***	**OR (95%CI)**	***p***
**Gender**										
Female	1.76 (1.26–2.47)	**0.001**	1.68 (1.29–2.20)	**< 0.001**	2.49 (1.90–3.26)	**< 0.001**	1.75 (1.31–2.34)	**< 0.001**	2.03 (1.55–2.67)	**< 0.001**
Male	Reference		Refe;rence		Reference		Reference		Reference	
**Age (years)**										
18–29	Reference		Reference		Reference		Reference		Reference	
30–39	1.07 (0.67–1.71)	0.9	1.61 (1.05–2.47)	**0.03**	1.14 (0.75–1.75)	0.5	0.78 (0.49–1.25)	0.3	1.57 (1.03–2.41)	**0.04**
40–49	1.98 (1.14–3.45)	**0.02**	1.70 (1.06–2.73)	**0.03**	1.48 (0.92–2.38)	0.1	0.72 (0.43–1.22)	0.2	2.40 (1.49–3.86)	**< 0.001**
50–59	3.06 (1.68–5.57)	**< 0.001**	1.86 (1.15–3.00)	**0.01**	1.99 (1.22–3.23)	**0.006**	1.12 (0.67–1.87)	0.7	3.43 (2.10–5.62)	**< 0.001**
60+	3.85 (1.91–7.78)	**< 0.001**	1.78 (1.03–3.05)	**0.04**	1.86 (1.08–3.22)	**0.03**	1.20 (0.68–2.13)	0.5	3.93 (2.25–6.86)	**< 0.001**
**Having higher education**										
Yes	1.43 (1.02–2.01)	**0.04**	1.45 (1.11–1.89)	**0.007**	1.53 (1.16–2.02)	**0.002**	1.39 (1.04–1.85)	**0.03**	1.23 (0.93–1.62)	0.1
No	Reference		Reference		Reference		Reference		Reference	
**Marital status**										
Single	Reference		Reference		Reference		Reference		Reference	
Married	0.85 (0.49–1.49)	0.6	0.75 (0.47–1.18)	0.2	0.71 (0.44–1.13)	0.1	0.90 (0.54–1.49)	0.7	0.95 (0.59–1.52)	0.8
Informal relationship	1.06 (0.62–1.81)	0.8	0.74 (0.46–1.17)	0.2	0.80 (0.51–1.27)	0.3	1.08 (0.66–1.77)	0.8	1.21 (0.77–1.93)	0.4
divorced/widowed	1.38 (0.62–3.09)	0.4	0.71 (0.40–1.26)	0.2	0.54 (0.30–1.00)	0.05	0.75 (0.40–1.41)	0.4	0.75 (0.41–1.37)	0.3
**Having children**										
Yes	1.01 (0.63–1.63)	0.9	0.97 (0.66–1.42)	0.9	1.25 (0.85–1.83)	0.3	1.22 (0.80–1.85)	0.4	1.08 (0.73–1.59)	0.7
No	Reference		Reference		Reference		Reference		Reference	
**Place of residence**										
Rural	Reference		Reference		Reference		Reference		Reference	
City below 20,000 residents	1.18 (0.65–2.12)	0.6	1.12 (0.72–1.73)	0.6	1.18 (0.75–1.85)	0.5	1.02 (0.63–1.65)	0.9	0.89 (0.57–1.40)	0.6
City from 20,000 to 99,999 residents	0.69 (0.45–1.05)	0.08	1.24 (0.87–1.77)	0.2	1.12 (0.78–1.61)	0.5	1.55 (1.06–2.27)	**0.02**	1.00 (0.69–1.43)	0.9
City from 100,000 to 499,999 residents	1.00 (0.61–1.78)	0.9	1.34 (0.92–1.95)	0.1	1.14 (0.78–1.67)	0.5	1.23 (0.82–1.85)	0.3	0.91 (0.62–1.33)	0.6
City above 500,000 residents	1.04 (0.61–1.78)	0.9	1.48 (0.98–2.23)	0.06	1.09 (0.72–1.67)	0.7	1.26 (0.81–1.97)	0.3	1.16 (0.76–1.79)	0.5
**Number of household members**										
1	0.72 (0.42–1.24)	0.2	0.95 (0.61–1.49)	0.8	1.36 (0.86–2.16)	0.2	1.15 (0.71–1.87)	0.6	1.25 (0.79–1.98)	0.3
2 or more	Reference		Reference		Reference		Reference		Reference	
**Occupational status**										
Active	1.15 (0.78–1.70)	0.5	1.06 (0.77–1.45)	0.7	0.97 (0.71–1.34)	0.9	1.19 (0.85–1.68)	0.3	1.10 (0.80–1.52)	0.6
Passive	Reference		Reference		Reference		Reference		Reference	
**Self–reported financial situation**										
Good	1.30 (0.85–1.99)	0.2	1.30 (0.93–1.83)	0.1	0.97 (0.68–1.37)	0.8	1.22 (0.85–1.77)	0.3	1.40 (0.99–1.99)	0.05
Moderate	1.10 (0.72–1.67)	0.7	1.01 (0.72–1.41)	0.9	0.99 (0.70–1.40)	0.9	0.91 (0.63–1.32)	0.6	1.20 (0.85–1.70)	0.3
Bad	Reference		Reference		Reference		Reference		Reference	
**Having diabetes**										
Yes	1.48 (0.77–2.86)	0.2	2.03 (1.33–3.1!)	**0.001**	2.29 (1.41–3.72)	**< 0.001**	1.89 (1.22–2.92)	**0.004**	1.31 (0.83–2.08)	0.3
No	Reference		Reference		Reference		Reference		Reference	
**History of diabetes in the family**										
Yes	1.62 (1.15–2.28)	**0.005**	1.50 (1.16–1.95)	**0.002**	1.66 (1.27–2.17)	**< 0.001**	1.56 (1.18–2.06)	**0.002**	2.04 (1.55–2.68)	**< 0.001**
No	Reference		Reference		Reference		Reference		Reference	
**Gender**										
Female	1.32 (1.01–1.72)	**0.04**	0.98 (0.73–1.33)	0.9	1.91 (1.39–2.64)	**< 0.001**	2.14 (1.52–3.01)	**< 0.001**	2.10 (1.55–2.84)	**< 0.001**
Male	Reference		Reference		Reference		Reference		Reference	
**Age (years)**										
18–29	Reference		Reference		Reference		Reference		Reference	
30–39	1.52 (0.99–2.32)	0.05	0.97 (0.61–1.53)	0.9	1.27 (0.77–2.12)	0.4	0.95 (0.53–1.70)	0.9	1.13 (0.71–1.78)	0.6
40–49	1.94 (1.21–3.09)	**0.006**	0.76 (0.44–1.28)	0.3	1.06 (0.74–2.33)	0.9	1.24 (0.67–2.29)	0.5	1.82 (1.08–3.07)	**0.02**
50–59	2.66 (1.65–4.29)	**< 0.001**	0.97 (0.57–1.63)	0.9	1.32 (0.74–2.33)	0.3	2.07 (1.14–3.75)	**0.02**	2.11 (1.23–3.62)	**0.007**
60+	2.21 (1.29–3.79)	**0.004**	0.59 (0.32–1.09)	0.09	1.31 (0.69–2.47)	0.4	1.47 (0.75–2.88)	0.3	1.37 (0.75–2.50)	0.3
**Having higher education**										
Yes	1.63 (1.25–2.13)	**< 0.001**	1.69 (1.25–2.28)	**< 0.001**	2.31 (1.68–3.16)	**< 0.001**	1.69 (1.22–2.35)	**0.002**	1.28 (0.95–1.74)	0.1
No	Reference		Reference		Reference		Reference		Reference	
**Marital status**										
Single	Reference		Reference		Reference		Reference		Reference	
Married	0.90 (0.60–1.42)	0.7	0.68 (0.41–1.14)	0.1	1.09 (0.62–1.89)	0.8	0.88 (0.49–1.59)	0.7	0.84 (0.50–1.40)	0.5
Informal relationship	1.08 (0.68–1.69)	0.8	0.93 (0.57–1.51)	0.8	0.96 (0.55–1.69)	0.9	0.70 (0.38–1.30)	0.3	1.00 (0.61–1.65)	0.9
divorced/widowed	0.87 (0.49–1.55)	0.6	1.11 (0.59–2.10)	0.7	0.71 (0.36–1.40)	0.3	0.76 (0.38–1.52)	0.4	0.76 (0.39–1.48)	0.4
**Having children**										
Yes	1.01 (0.69–1.47)	0.9	1.01 (0.66–1.55)	0.9	1.28 (0.75–2.17)	0.4	1.12 (0.69–1.81)	0.7	1.26 (0.82–1.92)	0.3
No	Reference		Reference		Reference		Reference		Reference	
**Place of residence**										
Rural	Reference		Reference		Reference		Reference		Reference	
City below 20,000 residents	0.93 (0.60–1.45)	0.8	0.90 (0.54–1.52)	0.7	1.18 (0.69–2.01)	0.6	1.50 (0.88–2.56)	0.1	0.90 (0.55–1.49)	0.7
City from 20,000 to 99,999 residents	1.04 (0.73–1.49)	0.8	1.63 (1.10–2.42)	**0.02**	1.50 (0.98–2.31)	0.06	1.69 (1.09–2.62)	**0.02**	1.14 (0.76–1.71)	0.5
City from 100,000 to 499,999 residents	1.08 (0.75–1.57)	0.7	1.13 (0.73–1.74)	0.6	1.74 (1.12–2.70)	**0.01**	1.13 (0.69–1.83)	0.6	0.91 (0.60–1.39)	0.7
City above 500,000 residents	0.86 (0.57–1.30)	0.5	1.95 (1.24–3.05)	**0.004**	1.56 (0.96–2.54)	0.08	1.23 (0.73–2.07)	0.4	1.23 (0.76–1.98)	0.4
**Number of household members**										
1	0.93 (0.60–1.45)	0.7	0.88 (0.54–1.43)	0.6	1.28 (0.75–2.17)	0.4	1.25 (0.72–2.16)	0.4	1.20 (0.72–1.98)	0.5
2 or more	Reference		Reference		Reference		Reference		Reference	
**Occupational status**										
Active	1.08 (0.79–1.47)	0.6	0.88 (0.62–1.25)	0.5	1.04 (0.71–1.52)	0.8	0.94 (0.63–1.38)	0.7	0.85 (0.59–1.22)	0.4
Passive	Reference		Reference		Reference		Reference		Reference	
**Self–reported financial situation**										
Good	1.25 (0.89–1.76)	0.2	1.09 (0.74–1.61)	0.7	1.12 (0.74–1.70)	0.6	0.83 (0.54–1.27)	0.4	1.33 (0.91–1.94)	0.1
Moderate	1.20 (0.86–1.68)	0.3	1.17 (0.80–1.71)	0.4	1.16 (0.78–1.75)	0.5	0.89 (0.59–1.34)	0.6	1.30 (0.89–1.90)	0.2
Bad	Reference		Reference		Reference		Reference		Reference	
**Having diabetes**										
Yes	2.52 (1.59–4.00)	**< 0.001**	2.43 (1.55–3.81)	**< 0.001**	1.18 (0.72–1.94)	0.5	1.26 (0.77–2.08)	0.4	0.92 (0.56–1.51)	0.7
No	Reference		Reference		Reference		Reference		Reference	
**History of diabetes in the family**										
Yes	1.84 (1.42–2.39)	**< 0.001**	1.72 (1.29–2.31)	**< 0.001**	1.50 (1.11–2.04)	**0.009**	1.38 (1.00–1.91)	0.5	1.91 (1.40–2.60)	**< 0.001**
No	Reference		Reference		Reference		Reference		Reference	

The bold values present results that meet the statistical significance requirement set at *p* < 0.05.

### 3.4. Factors associated with respondents' awareness of diabetes risk factors

Females were more likely (*p* < 0.05) to indicate overweight/obesity, low physical activity level, unhealthy diet, and genetic predisposition as diabetes risk factors (Table 8). Respondents over 40 years were more likely to indicate overweight/obesity, unhealthy diet, and genetic predisposition as diabetes risk factors (*p* < 0.05). Respondents with higher education were more aware of diabetes risk factors (*p* < 0.05). Respondents who had children were more likely to indicate overweight/obesity as a diabetes risk factor (*p* = 0.04). Respondents who lived alone were less likely to indicate excessive alcohol consumption as a diabetes risk factor (*p* = 0.02). Occupationally active individuals were more likely to indicate excessive alcohol consumption as a diabetes risk factor (*p* = 0.03). Respondents with a good financial situation were more likely to indicate overweight/obesity and an unhealthy diet as diabetes risk factors. General, respondents with a history of diabetes in the family had a higher level of knowledge of diabetes symptoms (Table 8). In the multivariable logistic regression model, there was no influence (*p* > 0.05) of the place of residence and health status (having diabetes) on the respondents' awareness of diabetes symptoms.

**Table 8:** Factors associated with awareness of risk factors for diabetes among adults in Poland (*n* = 1,051)—multivariable logistic regression model.

	**Factors associated with awareness of risk factors for diabetes among adults in Poland**											
**Variable**	**Excessive alcohol consumption**		**Smoking cigarettes/tobacco**		**Overweight/obesity**		**Low physical activity level**		**Unhealthy diet**		**Genetic Predisposition**	
	**OR (95%CI)**	***p***	**OR (95%CI)**	***p***	**OR (95%CI)**	***p***	**OR (95%CI)**	***p***	**OR (95%CI)**	***p***	**OR (95%CI)**	***p***
**Gender**												
Female	0.91 (0.69–1.20)	0.5	1.26 (0.93–1.72)	0.1	1.50 (1.07–2.09)	**0.02**	1.57 (1.20–2.06)	**< 0.001**	1.94 (1.44–2.62)	**< 0.001**	3.11 (2.31–4.18)	**< 0.001**
Male	Reference		Reference		Reference		Reference		Reference		Reference	
**Age (years)**												
18–29	Reference		Reference		Reference		Reference		Reference		Reference	
30–39	1.24 (0.80–1.92)	0.3	0.75 (0.45–1.23)	0.3	1.24 (0.76–2.01)	0.4	1.22 (0.79–1.88)	0.4	1.37 (0.86–2.17)	0.2	1.36 (0.86–2.13)	0.2
40–49	1.18 (0.73–1.92)	0.5	0.85 (0.50–1.45)	0.6	1.80 (1.04–3.12)	**0.04**	0.90 (0.56–1.43)	0.6	1.66 (0.99–2.77)	0.05	1.74 (1.05–2.87)	**0.03**
50–59	1.13 (0.69–1.86)	0.6	0.98 (0.57–1.67)	0.9	5.03 (2.63–9.60)	**< 0.001**	1.48 (0.91–2.39)	0.1	1.86 (1.10–3.15)	**0.02**	2.85 (1.67–4.86)	**< 0.001**
60+	1.02 (0.57–1.80)	0.9	0.68 (0.36–1.27)	0.2	2.39 (1.22–4.71)	**0.01**	1.09 (0.63–1.88)	0.8	2.12 (1.14–3.92)	**0.02**	1.99 (1.10–3.60)	**0.02**
**Having higher education**												
Yes	1.24 (0.94–1.64)	0.1	1.78 (1.31–2.43)	**< 0.001**	2.58 (1.80–3.70)	**< 0.001**	2.19 (1.66–2.88)	**< 0.001**	1.66 (1.22–2.26)	**0.001**	1.44 (1.07–1.94)	**0.02**
No	Reference		Reference		Reference		Reference		Reference		Reference	
**Marital status**												
Single	Reference		Reference		Reference		Reference		Reference		Reference	
Married	0.86 (0.53–1.40)	0.5	1.06 (0.62–1.83)	0.8	0.40 (0.22–0.70)	**0.001**	0.95 (0.59–1.51)	0.8	0.52 (0.31–0.88)	**0.01**	0.92 (0.56–1.53)	0.8
Informal relationship	1.08 (0.68–1.73)	0.7	1.11 (0.65–1.89)	0.7	0.81 (0.47–1.40)	0.4	1.02 (0.64–1.62)	0.9	0.88 (0.53–1.47)	0.6	1.08 (0.66–1.76)	0.8
divorced/widowed	1.21 (0.65–2.26)	0.5	0.81 (0.40–1.64)	0.6	0.53 (0.25–1.13)	0.1	1.08 (0.60–1.94)	0.8	0.83 (0.42–1.64)	0.6	0.78 (0.40–1.49)	0.4
**Having children**												
Yes	1.03 (0.69–1.54)	0.9	1.36 (0.87–2.14)	0.2	1.61 (1.02–2.55)	**0.04**	1.03 (0.70–1.51)	0.9	1.51 (0.99–2.29)	0.05	0.98 (0.65–1.49)	0.9
No	Reference		Reference		Reference		Reference		Reference		Reference	
**Place of residence**												
Rural	Reference		Reference		Reference		Reference		Reference		Reference	
City below 20,000 residents	1.33 (0.85–2.08)	0.2	1.22 (0.75–2.00)	0.4	0.81 (0.47–1.40)	0.4	0.96 (0.61–1.50)	0.9	0.74 (0.45–1.21)	0.2	0.99 (0.62–1.62)	0.9
City from 20,000 to 99,999 residents	0.95 (0.65–1.38)	0.8	0.84 (0.55–1.29)	0.4	0.81 (0.53–1.25)	0.3	1.06 (0.74–1.52)	0.8	0.96 (0.64–1.43)	0.8	1.34 (0.91–1.98)	0.2
City from 100,000 to 499,999 residents	1.04 (0.70–1.54)	0.9	0.98 (0.63–1.52)	0.9	1.04 (0.64–1.67)	0.9	1.21 (0.83–1.77)	0.3	0.75 (0.49–1.14)	0.2	1.03 (0.69–1.55)	0.9
City above 500,000 residents	1.16 (0.75–1.78)	0.5	1.32 (0.83–2.11)	0.2	1.30 (0.75–2.26)	0.4	1.32 (0.86–2.02)	0.2	1.03 (0.64–1.67)	0.9	1.60 (0.99–2.57)	0.05
**Number of household members**												
1	0.55 (0.39–0.90)	**0.02**	0.82 (0.47–1.42)	0.5	0.65 (0.37–1.13)	0.1	0.70 (0.45–1.10)	0.1	0.60 (0.37–1.00)	0.05	1.05 (0.64–1.72)	0.8
2 or more	Reference		Reference		Reference		Reference		Reference		Reference	
**Occupational status**												
Active	1.45 (1.04–2.03)	**0.03**	1.27 (0.87–1.84)	0.2	0.75 (0.50–1.12)	0.2	0.93 (0.67–1.28)	0.6	0.78 (0.55–1.12)	0.2	0.85 (0.60–1.21)	0.4
Passive	Reference		Reference		Reference		Reference		Reference		Reference	
**Self–reported financial situation**												
Good	0.91 (0.64–1.30)	0.6	0.68 (0.45–0.99)	**0.047**	1.67 (1.09–2.54)	**0.02**	0.91 (0.64–1.29)	0.6	1.57 (1.07–2.31)	**0.02**	1.22 (0.84–1.78)	0.3
Moderate	0.87 (0.61–1.24)	0.5	0.97 (0.67–1.43)	0.9	1.34 (0.89–2.02)	0.2	0.89 (0.63–1.25)	0.5	1.03 (0.71–1.49)	0.9	0.97 (0.67–1.40)	0.9
Bad	Reference		Reference		Reference		Reference		Reference		Reference	
**Having diabetes**												
Yes	1.07 (0.68–1.69)	0.8	0.70 (0.41–1.20)	0.2	1.83 (0.96–3.47)	0.07	1.45 (0.92–2.29)	0.1	1.19 (0.71–2.00)	0.5	1.18 (0.72–1.94)	0.5
No	Reference		Reference		Reference		Reference		Reference		Reference	
**History of diabetes in the family**												
Yes	1.44 (1.09–1.89)	**0.01**	1.18 (0.87–1.60)	0.3	1.32 (0.94–1.84)	0.1	1.75 (1.33–2.29)	**< 0.001**	1.60 (1.18–2.17)	**0.003**	2.08 (1.54–2.80)	**< 0.001**
No	Reference		Reference		Reference		Reference		Reference		Reference	

The bold values present results that meet the statistical significance requirement set at *p* < 0.05.

## 4. Discussion

To the authors' best knowledge, this is the most up-to-date study on the public awareness of diabetes among adults in Poland. This study revealed a limited level of public awareness of diabetes. The percentage of respondents who declared a lack of knowledge or little knowledge about diabetes was more than double the percentage of respondents who reported having good or rather good knowledge about this disease. Out of 10 symptoms of diabetes analyzed in this study, just half of them were correctly indicated by more than 50% of the respondents. Less than a quarter of respondents were able to point out such symptoms as increased risk of infections and persistent skin itching. Most of the respondents were able to correctly point overweight/obesity, unhealthy diet, and genetic predisposition as risk factors for diabetes, while excessive alcohol consumption, arterial hypertension, and being over 40–45 years old were recognized by less than one-third of respondents. Tobacco use was the least recognized diabetes risk factor. Respondents were also able to correctly identify most of the complications caused by diabetes, as well as preventive measures. Public awareness of selected aspects of diabetes varied by sociodemographic factors, of which gender, age, and educational level were the most important.

According to the review conducted by Gautam and Gupta knowledge is considered a key element in the control of diabetes mellitus epidemics (32). However, data on public awareness of diabetes are limited (33–36). Most recently published articles refer to studies conducted in developing countries such as India (33), Pakistan (34), Jordan (35), and Kenya (36). In contrary to this study, the abovementioned studies were carried out among respondents already diagnosed with diabetes or healthcare workers – not the general population (33–36). In Poland, the most recent available study on public awareness of diabetes was conducted in 2017 by Sobierajski (37). According to a 2017 study, general knowledge about risk factors, symptoms, and complications of diabetes in Poland was low. In 2017, only two (high blood sugar level, feeling sleepy) out of 16 symptoms of diabetes analyzed in the study, two out of 18 complications (diabetic coma, diabetic foot), and one out of 12 risk factors (overweight/obesity) were correctly identified by more than a half of respondents (37). When compared to 2017, findings from our study suggest that the level of public awareness of diabetes in Poland has increased. Nevertheless, significant gaps in public awareness of diabetes in Poland still exist, especially related to awareness of diabetes risk factors.

Awareness of symptoms of diabetes is crucial to early detection of the disease. However, the current study revealed a low level of awareness of major symptoms of diabetes in the general population in Poland. High blood glucose remained the most recognizable symptom of diabetes, as was pointed out by over 80% of respondents. This is a significant change compared to the 2017 study by Sobierajski (37) in which this symptom was identified by 56.5% of respondents. Other symptoms were indicated by a comparable percentage of respondents in 2017 and the current study. High blood glucose was also the most recognized symptom of diabetes indicated in studies carried out in developing countries (33–36). In this study, older respondents (aged 50 and over) were over three times more likely than younger respondents to indicate high blood glucose as a symptom of diabetes. Better knowledge of disease symptoms among older people is contrary to a study by Sørensen et al., who observed a decreasing health literacy with the age (38).

In this study, females, those with higher education, respondents diagnosed with diabetes as well as those with a history of diabetes in the family were more likely to correctly indicate symptoms of diabetes. This observation is in line with the study by Dos Santos et al. (39) (gender differences), and Kim et al. (40), who reported gender and educational differences in the level of public knowledge of diabetes. In this study, marital status, self-reported financial situation, and occupational status had no significant influence on public awareness of symptoms of diabetes. This is contrary to findings by Duplaga, who identified that health literacy in Poland was related to age, marital and vocational status (41).

A healthy lifestyle pattern is a well-known factor associated with decreased risk for diabetes, especially type 2 diabetes (42). Our study showed that knowledge about risk factors of diabetes in Poland is insufficient and unevenly distributed. Most of the respondents were able to point out overweight/obesity, unhealthy diet, and genetic predisposition as diabetes risk factors. Females and respondents over 40 years were significantly (up to three times) more likely to indicate these risk factors than other respondents. Having a higher education also influenced the public awareness of risk factors of diabetes (except for excessive alcohol consumption). As over 25% of Poles aged 15 and over are daily smokers and alcohol dependency remains one of the key problems in Poland, the public awareness of tobacco and alcohol use as a risk factor for diabetes is very limited (28).

Out of 11 different factors analyzed in this study, the number of household members, occupational status, and history of diabetes in the family were significantly associated with a higher level of awareness of excessive alcohol consumption as a diabetes risk factor. The number of household members and educational level were the only factors significantly associated with a higher level of awareness of tobacco smoking as a diabetes risk factor. In this study, a high level of awareness of overweight/obesity and unhealthy diet as a risk factor for diabetes may result from extensive campaigns on di-et-related diseases that were carried out in Poland in recent years (43). We can hypothesize that a low level of awareness of alcohol and tobacco consumption as a risk factor for diabetes may result from a relatively low number of educational campaigns on diabetes risk factors or its limited effectiveness. Particular attention should be paid to males who are at higher risk of substance use and presented a lower level of aware-ness of diabetes risk factors, especially alcohol and tobacco use.

Findings from this study on awareness of diabetes prevention methods reflect the knowledge of respondents about its risk factors. The most recognized diabetes prevention methods were limited consumption of carbohydrates (sugars) in the diet, weight reduction, and regular physical activity. A higher level of awareness of diabetes prevention methods was associated with higher age and educational level, as well as being married and having children.

It is believed that effective diabetes education can minimize the risk of long-term diabetes complications (44). Findings from this study show that only the most visible complications of this disease (diabetes foot, limb amputation) were widely recognized by adults in Poland. This finding corresponds with a high rate of lower limb amputations performed in Poland (approx. 7–8 thousand each year) of which over a half is performed in diabetic patients (1.7 per 1,000 patients diagnosed with diabetes) (45). This study showed a low level of awareness of diabetes-related nephropathy or neuropathy among adults in Poland. This finding underlines the need to increase the level of public awareness of long-term diabetes-related complications, especially those which do not show any visible symptoms for many years. As in the case of risk factors, symptoms, and prevention methods, awareness of diabetes-related complications was significantly associated with female gender, older age, and higher education level.

Out of 11 sociodemographic factors analyzed in this study, gender and education-al level were the most important factors significantly associated with a higher level of general knowledge on diabetes. In this study older age was associated with better knowledge about the disease which is contrary to the study by Sørensen et al. (38). Findings from this study also showed, that having a person with diabetes in the family leads to a better understanding of this condition. We can hypnotize that this is due to a specific character of diabetes – as a chronic disease, that manifests in older age and the patient often requires family support and engagement in disease management. These may supplement, but should not substitute a proper diabetic education, that should be provided as a part of a public health intervention on diabetes. In this study, diagnosis of diabetes had a limited impact on the level of knowledge on diabetes (two out of six questions on complications and none of the questions on prevention methods), so we can hypothesize that the effectiveness of currently available educational activities targeted to patients with diabetes is limited and requires further improvements.

This study has numerous practical implications for public health interventions in Poland. It reveals an insufficient level of public awareness of diabetes, its risk factors, symptoms, and complications, as well as available preventive methods. This finding underlines a need to conduct a nationwide educational campaign on diabetes. Personalized communication should be targeted to younger individuals as well as males without higher education, as these groups were identified as those with the lowest level of awareness of diabetes. Moreover, this study indicates poor quality of education for patients already diagnosed with diabetes in Poland. General practitioners as well as internal medicine specialists and diabetologists should be actively involved in educational activities targeted to patients at higher risk of diabetes. Findings from this study also underline the positive influence of having a family member with diabetes on the level of awareness of diabetes among other family members. The COVID-19 pandemic has a negative impact on diabetes care in Poland (13, 46), so public health interventions aimed to increase the level of public awareness of diabetes are needed to reduce the diabetes burden in Poland. Further studies should analyze the impact of the health system and diabetes education provided by healthcare workers on public awareness of diabetes.

This study has some limitations. The study was carried out using the CAWI re-search method, which excludes the direct interaction of the interviewer with the respondent (e.g., the ability to assess the competencies of the respondents, and her/his ability to understand the questions asked). The study questionnaire was limited to the most prevalent symptoms, risk factors, and complications. History of diabetes (both diagnosed by a doctor and diabetes in the family) was self-declared, and medical records were not verified due to the study design. Moreover, this research method includes only subjects who have internet access (though more than 92% of households in Poland now have internet access) (47). Nevertheless, this is the most comprehensive and up-to-date study on public knowledge and awareness of diabetes that was carried out among adults in Poland, after the COVID-19 pandemic outbreak.

## 5. Conclusions

This study demonstrated insufficient public awareness of diabetes among adults in Poland. Gender and educational level were the most important factors significantly associated with the awareness of the selected aspects of diabetes, while self-reported financial situation and place of residence had none or marginal influence. Moreover, the current study indicated significant gaps in the knowledge about risk factors for diabetes and its complications, as well as methods to prevent them. The presented data manifest the importance of adopting a comprehensive education strategy regarding diabetes in Poland.

## Data availability statement

The raw data supporting the conclusions of this article will be made available by the authors, without undue reservation.

## Ethics statement

The study protocol was reviewed and approved by the Ethical Review Board at the Centre of Postgraduate Medical Education, Warsaw, Poland (No. 70/2022; date of approval: 08 June 2022). The patients/participants provided their written informed consent to participate in this study.

## Author contributions

KS: conceptualization, data curation, formal analysis, investigation, project administration, visualisation, and writing an original draft. JG-S: conceptualization, investigation, methodology, and manuscript review and editing. JP: conceptualization, supervision, and manuscript review and editing. MJ: conceptualization, formal analysis, and manuscript review and editing. All authors contributed to the article and approved the submitted version.

## References

[1] Standl E Khunti K Hansen TB Schnell O. The global epidemics of diabetes in the 21st century: Current situation and perspectives. Eur J Prev Cardiol. (2019) 26:7–14. 10.1177/2047487319881021 31766915

[2] Saeedi P Petersohn I Salpea P Malanda B Karuranga S Unwin N. Global and regional diabetes prevalence estimates for 2019 and projections for 2030 and 2045: Results from the International Diabetes Federation Diabetes Atlas, 9th edition. Diabetes Res Clin Pract. (2019) 157:107843. 10.1016/j.diabres.2019.107843 31518657

[3] Cho NH Shaw JE Karuranga S Huang Y da Rocha Fernandes JD Ohlrogge AW. IDF Diabetes Atlas: Global estimates of diabetes prevalence for 2017 and projections for 2045. Diabetes Res Clin Pract. (2018) 138:271–81. 10.1016/j.diabres.2018.02.023 29496507

[4] World Health Organization. Noncommunicable Diseases Progress Monitor 2020 (2022). Available online at: https://www.who.int/publications/i/item/9789240000490 (accessed August 25, 2022).

[5] Banday MZ Sameer AS Nissar S. Pathophysiology of diabetes: An overview. Avicenna J Med. (2020) 10:174–88. 10.4103/ajm.ajm_53_20 33437689 PMC7791288

[6] Tao Z Shi A Zhao J. Epidemiological Perspectives of Diabetes. Cell Biochem Biophys. (2015) 73:181–5. 10.1007/s12013-015-0598-4 25711186

[7] Xu G Liu B Sun Y Du Y Snetselaar LG Hu FB. Prevalence of diagnosed type 1 and type 2 diabetes among US adults in 2016 and 2017: population based study. BMJ. (2018) 362:k1497. 10.1136/bmj.k1497 30181166 PMC6122253

[8] The International Diabetes Federation. IDF Diabetes Atlas (2022). Available online at: https://diabetesatlas.org/ (accessed August 25, 2022).

[9] Chen L Magliano DJ Zimmet PZ. The worldwide epidemiology of type 2 diabetes mellitus–present and future perspectives. Nat Rev Endocrinol. (2011) 8:228–36. 10.1038/nrendo.2011.183 22064493

[10] Lovic D Piperidou A Zografou I Grassos H Pittaras A Manolis A. The Growing Epidemic of Diabetes Mellitus. Curr Vasc Pharmacol. (2020) 18:104–9. 10.2174/1570161117666190405165911 30961501

[11] NCD Risk Factor Collaboration (NCD-RisC). Worldwide trends in diabetes since 1980: a pooled analysis of 751 population-based studies with 4.4 million participants. Lancet. (2016) 387:1513–30. 10.1016/S0140-6736(16)00618-8 27061677 PMC5081106

[12] Kolb H Martin S. Environmental/lifestyle factors in the pathogenesis and prevention of type 2 diabetes. BMC Med. (2017) 15:131. 10.1186/s12916-017-0901-x 28720102 PMC5516328

[13] Grudziaz-Sekowska J Sekowski K Kobuszewski B. Healthcare Utilization and Adherence to Treatment Recommendations among Children with Type 1 Diabetes in Poland during the COVID-19 Pandemic. Int J Environ Res Public Health. (2022) 19:4798. 10.3390/ijerph19084798 35457665 PMC9031476

[14] Zatońska K Basiak-Rasała A Rózańska D Karczewski M Wołyniec M Szuba A. Changes in diabetes prevalence and corresponding risk factors - findings from 3- and 6-year follow-up of PURE Poland cohort study. BMC Public Health. (2020) 20:843. 10.1186/s12889-020-08970-5 32493306 PMC7268673

[15] OECD/European Union. Health at a Glance: Europe 2020: State of Health in the EU Cycle, OECD Publishing, Paris (2020). (accessed August 25, 2022).

[16] Ministry of Health of the Republic of Poland. Diabets in numbers (2022). Available online at: https://pacjent.gov.pl/artykul/cukrzyca-w-liczbach (accessed August 25, 2022).

[17] Topor-Madry R Wojtyniak B Strojek K Rutkowski D Bogusławski S Ignaszewska-Wyrzykowska A. Prevalence of diabetes in Poland: a combined analysis of national databases. Diabet Med. (2019) 36:1209–16. 10.1111/dme.13949 30889281

[18] Mularczyk-Tomczewska P Zarnowski A Gujski M Sytnik-Czetwertyński J Pańkowski I Smoliński R. Preventive health screening during the COVID-19 pandemic: A cross-sectional survey among 102,928 internet users in Poland. J Clin Med. (2022) 11:3423. 10.3390/jcm11123423 35743493 PMC9224829

[19] American Diabetes Association. Standards of medical care in diabetes−2013. Diabetes Care. (2013) 36:S11–66. 10.2337/dc13-S011 23264422 PMC3537269

[20] Funnell MM Anderson RM. MSJAMA the problem with compliance in diabetes. JAMA. (2000) 284:1709. 10.1001/jama.284.13.1709-JMS1004-6-1 11015809

[21] Waheedi M Awad A Hatoum HT Enlund H. The relationship between patients' knowledge of diabetes therapeutic goals and self-management behaviour, including adherence. Int J Clin Pharm. (2017) 39:45–51. 10.1007/s11096-016-0375-5 27878750

[22] Chavan GM Waghachavare VB Gore AD Chavan VM Dhobale RV Dhumale GB. Knowledge about diabetes and relationship between compliance to the management among the diabetic patients from Rural Area of Sangli District, Maharashtra, India. J Family Med Prim Care. (2015) 4:439–43. 10.4103/2249-4863.161349 26288789 PMC4535111

[23] Powers MA Bardsley J Cypress M Duker P Funnell MM Hess Fischl A. Diabetes self-management education and support in type 2 diabetes: A joint position statement of the american diabetes association, the American Association of diabetes educators, and the academy of nutrition and dietetics. Diabetes Care. (2015) 38:1372–82. 10.2337/dc15-0730 26048904

[24] Polish Diabetes Society. Clinical recommendations for the management of diabetes mellitus 2020 (2020). Available online at: https://ptmr.info.pl/wp-content/uploads/2021/03/Zalecenia-kliniczne-dotyczace-postepowania-u-chorych-na-cukrzyce-2020.pdf (accessed August 25, 2022).

[25] Preethikaa S Brundha MP. Awareness of diabetes mellitus among general population. Res J Pharm Technol. (2018) 11:1825–9. 10.5958/0974-360X.2018.00339.634812167

[26] Al Sayah F Majumdar SR Williams B Robertson S Johnson JA. Health literacy and health outcomes in diabetes: a systematic review. J Gen Intern Med. (2013) 28:444–52. 10.1007/s11606-012-2241-z 23065575 PMC3579965

[27] The Nationwide Research Panel Ariadna. About us (2022). Available online at: https://panelariadna.com/ (accessed August 25, 2022).

[28] Jankowski M Ostrowska A Sierpiński R Skowron A Sytnik-Czetwertyński J Giermaziak W. The prevalence of tobacco, heated tobacco, and e-cigarette use in Poland: A 2022 web-based cross-sectional survey. Int J Environ Res Public Health. (2022) 19:4904. 10.3390/ijerph19084904 35457771 PMC9031359

[29] Zarnowski A Jankowski M Gujski M. The use of mobile apps and wearables to monitor diet, weight, and physical activity – a cross-sectional survey among adults in Poland. Med Sci Monit. (2022) 28:e937948. 10.12659/MSM.937948 36081328 PMC9473310

[30] Garcia AA Villagomez ET Brown SA Kouzekanani K Hanis CL. The Starr County Diabetes Education Study: development of the Spanish-language diabetes knowledge questionnaire. Diabetes Care. (2001) 24:16–21. 10.2337/diacare.24.1.16 11194219

[31] Fitzgerald JT Funnell MM Hess GE Barr PA Anderson RM Hiss RG. The reliability and validity of a brief diabetes knowledge test. Diabetes Care. (1998) 21:706–10. 10.2337/diacare.21.5.706 9589228

[32] Gautam SK Gupta V. Impact of knowledge, attitude and practice on the management of type 2 diabetes mellitus in developing countries: a review. Curr Diabetes Rev. (2022) 18:e010521189965. 10.2174/1573399817666210106104230 33413065

[33] Murugesan N Snehalatha C Shobhana R Roglic G Ramachandran A. Awareness about diabetes and its complications in the general and diabetic population in a city in southern India. Diabetes Res Clin Pract. (2007) 77:433–7. 10.1016/j.diabres.2007.01.004 17291622

[34] Gillani AH Amirul Islam FM Hayat K Atif N Yang C Chang J. Knowledge, attitudes and practices regarding diabetes in the general population: a cross-sectional study from Pakistan. Int J Environ Res Public Health. (2018) 15:1906. 10.3390/ijerph15091906 30200534 PMC6164838

[35] Alsous M Abdel Jalil M Odeh M Al Kurdi R Alnan M. Public knowledge, attitudes and practices toward diabetes mellitus: A cross-sectional study from Jordan. PLoS ONE. (2019) 14:e0214479. 10.1371/journal.pone.0214479 30925187 PMC6440628

[36] Mohamed SF Mwangi M Mutua MK Kibachio J Hussein A Ndegwa Z. Prevalence and factors associated with pre-diabetes and diabetes mellitus in Kenya: results from a national survey. BMC Public Health. (2018) 18:1215. 10.1186/s12889-018-6053-x 30400865 PMC6218998

[37] Sobierajski T. Coalition for fight against diabetes. Report: social image of diabetes [Polish] (2017). Available online at: https://nazdrowie.pl/wp-content/uploads/2017/11/Spo%C5%82eczny-obraz-cukrzycy_raport.pdf (accessed August 25, 2022).

[38] Sørensen K Pelikan JM Röthlin F Ganahl K Slonska Z Doyle G. Health literacy in Europe: comparative results of the European health literacy survey (HLS-EU). Eur J Public Health. (2015) 25:1053–8. 10.1093/eurpub/ckv043 25843827 PMC4668324

[39] Lemes Dos Santos PF Dos Santos PR Ferrari GS Fonseca GA Ferrari CK. Knowledge of diabetes mellitus: does gender make a difference? Osong Public Health Res Perspect. (2014) 5:199–203. 10.1016/j.phrp.2014.06.004 25379370 PMC4215000

[40] Kim S Love F Quistberg DA Shea JA. Association of health literacy with self-management behavior in patients with diabetes. Diabetes Care. (2004) 27:2980–2. 10.2337/diacare.27.12.2980 15562219

[41] Duplaga M. Determinants and consequences of limited health literacy in polish society. Int J Environ Res Public Health. (2020) 17:642. 10.3390/ijerph17020642 31963834 PMC7014389

[42] Bellou V Belbasis L Tzoulaki I Evangelou E. Risk factors for type 2 diabetes mellitus: An exposure-wide umbrella review of meta-analyses. PLoS ONE. (2018) 13:e0194127. 10.1371/journal.pone.0194127 29558518 PMC5860745

[43] Zarnowski A Jankowski M Gujski M. Public Awareness of Diet-Related Diseases and Dietary Risk Factors: A 2022 Nationwide Cross-Sectional Survey among Adults in Poland. Nutrients. (2022) 14:3285. 10.3390/nu14163285 36014795 PMC9416498

[44] Nazar CM Bojerenu MM Safdar M Marwat J. Effectiveness of diabetes education and awareness of diabetes mellitus in combating diabetes in the United Kigdom; a literature review. J Nephropharmacol. (2015) 5:110–5. 28197516 PMC5297564

[45] Wierzba W Krasnodebski P Sliwczyński A Karnafel W. Geographic variability of major non-traumatic lower limb amputations in diabetic and non-diabetic patients in Poland. Ann Agric Environ Med. (2020) 27:76–9. 10.26444/aaem/114725 32208583

[46] Sekowski K Grudziaz-Sekowska J Goryński P Pinkas J Jankowski M. Epidemiological Analysis of Diabetes-Related Hospitalization in Poland before and during the COVID-19 Pandemic, 2014–2020. Int J Environ Res Public Health. (2022) 19:10030. 10.3390/ijerph191610030 36011665 PMC9407838

[47] Central Statistical Office. Information Society in Poland in 2020 (2021). Available online at: https://stat.gov.pl/obszary-tematyczne/nauka-i-technika-spoleczenstwo-informacyjne/spoleczenstwo-informacyjne/spoleczenstwo-informacyjne-w-polsce-w-2020-roku,2,10.html (accessed August 25, 2022).

